# SGLT2-inhibitors and euglycemic diabetic ketoacidosis in COVID-19 pandemic era: a case report

**DOI:** 10.1007/s00592-022-01909-9

**Published:** 2022-06-19

**Authors:** Enzo Secinaro, Simone Ciavarella, Giulia Rizzo, Ettore Porreca, Ester Vitacolonna

**Affiliations:** 1 Department of Innovative Technologies in Medicine and Dentistry, School of Medicine, and Health Sciences, “G. D’Annunzio” University of Chieti-Pescara, Chieti, Italy; 2 Division of Internal Medicine, Department of Internal Medicine, SS Annunziata Hospital-Chieti, Chieti, Italy; 3 Diabetes, Nutrition and Metabolic Diseases Unit, SS Annunziata Hospital-Chieti, Chieti, Italy; 4 Department of Medicine and Ageing Sciences, “G. D’ Annunzio” University of Chieti-Pescara, Chieti, Italy

**Keywords:** SGLT2-inhibitors, Euglycemic diabetic ketoacidosis, COVID-19, Low-carbohydrate intake, LADA

## Introduction

According to the American Diabetes Association’s criteria, diabetic ketoacidosis (DKA) is a clinical condition defined with a pH ≤ 7.30, serum bicarbonate ≤ 18, and positive urine ketones. This severe metabolic complication is typically associated with Type 1 diabetes mellitus (T1DM), as a first presentation or as a consequence of non-optimal insulin therapy management; however, it can occasionally affect Type 2 diabetes mellitus (T2DM) patients. It is important to point out that diabetic ketoacidosis is usually associated with hyperglycemia, even though, it occasionally may occur with normal or slightly increased glycemia serum levels (< 200 mg/dl), as a condition known as euglycemic ketoacidosis (eDKA). As showed by relevant literature, this condition appears to be increasingly frequent after the introduction of sodium glucose-cotransporter 2 inhibitors (SGLT2i), a new class of drugs for the treatment of T2DM patients [1]. Interestingly, a recent meta-analysis has shown that in adults with T2DM, SGLT2-inhibitors were found to increase the risk of DKA in both observational studies and large randomized clinical trials [1]. The interest in this drug class has broadened due to the evidence of cardio and renal protection it offers to subjects with established cardiovascular disease. In May 2015, the Food and Drug Administration (FDA) warned that treatment with SGLT2i might increase the risk of euglycemic ketoacidosis, due to glycosuria induced by the specific inhibition of glucose reabsorption of in the renal tubule. DKA and eDKA have several predisposing factors in common: poor beta-cell function reserve, dehydration, reduced carbohydrate intake, counter-regulatory hormone action (steroids, glucagon, adrenergic hormones), and all type of stress agents (acute illness, cancer, pregnancy, starvation, surgery, and drugs). Among these stressors, SARS-CoV2 infection and COVID-19 have been of current concern since both conditions might lead, through different pathways, to ketogenesis, in particular in T2DM patients treated with SGLT2i.

The aim of this study is to present a case of eDKA in a patient with T2DM who was being treated with SGLT2i therapy and who was hospitalized for SARS-CoV2-related pneumonia. It is worth noticing that the role of COVID19 in the development of eDKA might have often underdiagnosed, thus leading to severe metabolic consequences. Another aim is to highlight the crucial role of the patient phenotyping [2] and education, in order to choose the best possible tailored therapy (including SGLT2i,) after assessing risks and benefits for the individual.

## Case report

A 54-year-old woman presented to our emergency department (ED) with fever, cough, and lamenting shortness of breath for the previous 2 days. The patient denied any history of alcohol or illicit drug use, diarrhea, vomiting, prolonged fasting, bleeding, or previous surgeries. Notably, our patient reported to physicians a very low-carbohydrate and food intake during the last days due to her critical clinical conditions. Her past medical history was remarkable due to rheumatoid arthritis. In addition, she had had T2DM without chronic complications for 5 years, for which she was being followed up at a Diabetes and Metabolic Diseases Center in another city. The patient reported that she was first treated with metformin therapy, which was then suspended due to intolerance. For this reason, she was subsequently advised to take empagliflozin and linagliptin. The last value of the patient’s HbA1c was 89 mmol/mol (10.29%). At admission to our clinic, SARS-CoV-2 RT- PCR tested positive. Chest CT excluded pulmonary embolism, however showing subpleural ground-glass opacification, mostly in the basal region of lung. The patient was admitted to the Medical unit for treatment. Vital signs at admission were blood pressure 145/90 mmHg, heart rate 95 beats/min, temperature 36.5 °C, respiratory rate 20 breaths/min. She was conscious and alert with no focal signs.

Physical examination revealed body weight of 73 kg, height 163 cm, BMI 27.5 kg/m^2^, oxygen saturation 91% with 4L/min supplementary O2 with Venturi mask. She was moderately dehydrated, with a dry oral mucosa and poor skin turgor. Lung examination revealed bilateral basilar crackles, no rales, nor wheezes. Cardiac and abdominal examinations were unremarkable.

Arterial blood gases showed metabolic acidosis with an elevated anion gap (pH 7.294, CO2 31 mm Hg, HCO3 18 mEq/L, BE -7.4, anion gap 20 mEq/L, lactate 1.14 mol/L), though glycemia was found to be mildly elevated (159 mg/dL). Serum electrolytes and creatinine were within standard. Urinalysis was positive for ketonic bodies. A suspicion of euglycemic diabetic ketoacidosis (eDKA) was posed.

As a first intervention, intravenous regular insulin was started (0.03 UI/kg/h) with normal saline (NaCl 0.9%) infusion. Potassium levels were closely monitored without any need for supplementation. Oral antidiabetic medications were discontinued.

After starting her therapy, the patient had clinical recovery, with an improvement of acidosis. Due to the patient’s history and her clinical presentation, considering that she was a carrier of an autoimmune disease and had been on steroid treatment for years, we did not exclude a Latent Autoimmune Diabetes in Adults (LADA) dosing anti-GAD, ICA e Ia2 antibodies, despite the autoantibodies had been found to be low concentration.

In addition, C-peptide was tested and resulted in the low-normal range of the scale (C-peptide 1.34 ng/ml). As for what regards the management of SARS-CoV-2 infection, non-invasive mechanical ventilation was needed for the first three days. Thromboembolic prophylaxis with Enoxaparin 4000 IU s.c. qd was administered.

After 14 days, the patient was discharged in good clinical status and prescribed treatment with multiple daily injection (MDI) insulin therapy (glargine and lispro).

## Discussion

Sodium glucose co-transporter 2 (SGLT-2) inhibitors are a pharmacologically novel class of agents used in the treatment of T2DM. In 2013, canagliflozin was the first SGLT-2 inhibitor approved for glycemic control in patients with T2DM, followed by the approval of dapagliflozin and empagliflozin. These agents achieve their glucose-lowering effect independent of insulin, via inhibition of the sodium glucose co-transporter 2, expressed in the proximal tubule within the kidney, increasing urinary excretion of glucose. This class of drugs produces a significant reduction in HbA1c, as well as weight loss, reduction in systolic and diastolic blood pressure via its diuretic effect and lower risk of hypoglycemia compared to insulin secretagogues. All these beneficial effects cause a reduction in major adverse cardiovascular events (MACEs) and delay progression of nephropathy in patients with clinically established cardiovascular disease, as demonstrated in CANVAS and EMPA-REG studies. These trials compared, respectively, empagliflozin and canagliflozin with placebo in patients affected by T2DM and with an elevated risk of cardiovascular disease, showing these drugs can produce not only a significant reduction in adverse cardiovascular outcomes (such as death from cardiovascular causes, nonfatal myocardial infarction, or non-fatal stroke), but also a significant reduction in hospitalization for heart failure [3]. Moreover, the American Diabetes Association and the National guidelines recommend that a SGLT-2 inhibitor with demonstrated cardiovascular benefit be used in patients with established atherosclerotic cardiac disease, established kidney disease, or established heart failure. The recent guidelines of the European Society of Cardiology (ESC) for heart failure also recommend the use of empagliflozin and dapagliflozin as first-line treatment for HFrEF (heart failure with reduced ejection fraction) regardless of whether patient suffers from diabetes, unless contraindicated or not tolerated [4].

Despite these benefits, SGLT-2 inhibitors have been associated with numerous adverse events such as genitourinary tract infections, volume depletion, and diabetic ketoacidosis (DKA), in particular euglycemic ketoacidosis, due in part to their mechanism of action [5].

In patients treated with SGLT2-inhibitors, DKA is precipitated by inadequate/discontinued insulin therapy, low-carbohydrate intake, prolonged fasting, alcohol consumption, severe infections/illness/surgery, and conditions which increase counter-regulatory hormone production (glucagon, catecholamines, cortisol, and growth hormone) [5]. Interestingly, recent studies show that in adults with T2DM, SGLT2-inhibitors were found to increase the risk of DKA in both observational studies and large randomized clinical trials [1].

Among these stressors, SARS-CoV2 infection and COVID-19 are of current concern since these conditions can lead, through different pathways, to ketogenesis, in particular in T2DM patients treated with SGLT2i.

Our patient presented several of these stressors: COVID-19, very low-carbohydrate and food intake due to her clinical condition,, dehydration, low BMI, endogenous and exogenous counter-regulatory hormones (catecholamines induced by illness and the treatment with steroids), and SGLT2i use as treatment of T2DM without a strong indication. The first-line treatment for our subject should have been metformin, since she did not present an established cardiovascular risk or a diagnosed cardiovascular disease such as heart failure, chronic kidney disease, or atherosclerosis. Our patient reported that she was first treated with metformin therapy, which was then suspended due to intolerance. There is a debate whether the “normal” glucose in eDKA may be partly derived from decreased endogenous glucose production via gluconeogenesis or from increased urinal loss.

Decreased carbohydrate intake can cause insulinopenia and increased glucagon levels. Increased glucagon/insulin ratio, in turn, further promotes lipolysis and ketogenesis. Meanwhile, carbohydrate deficit and continued insulin treatment facilitate euglycemia.

By competitive inhibition of SGLT-2 at the proximal convoluted tubule, SGLT-2 inhibitors block the reabsorption of 30 ~ 50% of filtered glucose from the primary urine. The hypoglycemic effect of this “carbohydrate deficit” is only partly offset by the increased endogenous glucose production (EGP) due to gluconeogenesis [5]. There is a metabolic shift from glucose utilization to lipid utilization, just as it happens in starvation. Lower blood glucose levels can cause a decrease in circulating insulin and an increase in glucagon concentration. The SGLT-2 inhibitor per se is a stimulator of glucagon secretion, which further enhances lipolysis and ketogenesis. Decreased reabsorption of ketones also contributes to ketonemia. Although there is no established phenotype in T2DM concerning SGLT-2 inhibitor-associated eDKA, it appears that patients with a longer history of diabetes, poorer control of disease, lower BMI, and poorer β cell function reserve are more susceptible to eDKA.

An increased incidence of new-onset diabetes and DKA has been seen during the COVID-19 pandemic. SARS-CoV2 infection and COVID-19 are of remarkable concern, since both conditions might lead, via different routes, to ketogenesis, in particular in T2DM patients treated with SGLT2i.

A direct toxic effect of the SARS-CoV-2 virus on pancreatic islets, as seen with SARS-CoV-1, could contribute to this condition. Pancreatic islets show an increased expression of angiotensin-converting enzyme 2 receptors, which may lead to a higher rate of cell death among insulin-producing cells, decreased endogenous insulin production, and increased likelihood of DKA. In addition, elevations in proinflammatory cytokines, such as interleukin 6, can be found in severe COVID-19 infections. This response may also contribute to ketoacidosis: interleukin 6 has been implicated in hyperglycemia induced by physiologic stress, and the elevation is even higher in patients with impaired glucose tolerance. Thus, a cytokine storm is another mechanism that may potentially contribute to an increased incidence of eDKA in patients with COVID-19 taking SGLT2i.

Steroids are considered the main treatment for many inflammatory, immunologic, allergic, and malignant diseases. Our patient was in chronic treatment with corticosteroids for rheumatoid arthritis, recently increased in dosage for SARS-Cov-2-related respiratory failure.

A major adverse effect of steroids is hyperglycemia, which can worsen pre-existing diabetes or precipitate new diabetes through multifactorial mechanisms, including increased levels of hepatic glucocorticoids, alteration of receptor function, glucose uptake inhibition in adipose tissue, and abnormal carbohydrate metabolism, which may lead to insulin resistance [5].

## Conclusions

Many cases report that therapy with SGLT2-inhibitors in T2DM may be related to an increase in the risk of DKA [1]. This case highlights the potential increased danger of SGLT2i use in an acute illness, such as COVID-19, even in patients who are not insulin-treated or have already discontinued the medication.

The medical community should keep in mind the possibility that drawbacks may outweigh the possible benefits of such treatment in diabetic SARS CoV-2-infected patient. For this reason, all patients taking SGLT2i should consider discontinuing the medication if they become ill or have poor carbohydrate intake. Blood glucose and ketones monitoring would be advisable for an early diagnosis and treatment of eDKA, even after the discontinuation of SGLT2i since the metabolic effects of these drugs could persist up to 24–48 h. In addition, our patient was affected by another autoimmune disease and had clinical features suggestive of possible LADA, despite the autoantibodies being negative.

In conclusion, considering the widespread use of SGLT2i for diabetic and non-diabetic patients (HFrEF) and the relevant risk of the adverse events described above, the prescription of this class of drugs should be restricted to trained physicians who can provide patients not only with customized treatment, but also with appropriate information and education in concerning situations. In order to optimize the efficacy and safety of sGLT2i therapy, it is essential that patients undergoing treatment are phenotyped, following current recommendations [2]. This will contribute to highlight the importance of patients’ education as well as to reduce the risk of adverse events, paving the way for tailored pharmacological treatment.
